# Polyphenol Metabolite Pyrogallol-*O*-Sulfate Decreases Microglial Activation and VEGF in Retinal Pigment Epithelium Cells and Diabetic Mouse Retina

**DOI:** 10.3390/ijms222111402

**Published:** 2021-10-22

**Authors:** Daniela F. Santos, Mariana Pais, Cláudia N. Santos, Gabriela A. Silva

**Affiliations:** 1 iNOVA4HEALTH, CEDOC, NOVA Medical School, Faculdade de Ciências Médicas, Universidade Nova de Lisboa, 1169-056 Lisboa, Portugal; daniela.filipa.santos@nms.unl.pt (D.F.S.); mariana.pais@nms.unl.pt (M.P.); claudia.nunes.santos@nms.unl.pt (C.N.S.); 2 ProRegeM PhD Programme—NOVA Medical School, Faculdade de Ciências Médicas, Universidade Nova de Lisboa, 1169-056 Lisboa, Portugal; 3 NOVA Medical School, Faculdade de Ciências Médicas, Universidade Nova de Lisboa, 1169-056 Lisboa, Portugal

**Keywords:** diabetic retinopathy, inflammation, neovascularization, retinal pigment epithelium, hyperglycemia

## Abstract

(Poly)phenol-derived metabolites are small molecules resulting from (poly)phenol metabolization after ingestion that can be found in circulation. In the last decade, studies on the impact of (poly)phenol properties in health and cellular metabolism accumulated evidence that (poly)phenols are beneficial against human diseases. Diabetic retinopathy (DR) is characterized by inflammation and neovascularization and targeting these is of therapeutic interest. We aimed to study the effect of pyrogallol-*O*-sulfate (Pyr-s) metabolite in the expression of proteins involved in retinal glial activation, neovascularization, and glucose transport. The expression of PEDF, VEGF, and GLUT-1 were analyzed upon pyrogallol-*O*-sulfate treatment in RPE cells under high glucose and hypoxia. To test its effect on a diabetic mouse model, Ins2^Akita^ mice were subjected to a single intraocular injection of the metabolite and the expression of PEDF, VEGF, GLUT-1, Iba1, or GFAP measured in the neural retina and/or retinal pigment epithelium (RPE), two weeks after treatment. We observed a significant decrease in the expression of pro-angiogenic VEGF in RPE cells. Moreover, pyrogallol-*O*-sulfate significantly decreased the expression of microglial marker Iba1 in the diabetic retina at different stages of disease progression. These results highlight the potential pyrogallol-*O*-sulfate metabolite as a preventive approach towards DR progression, targeting molecules involved in both inflammation and neovascularization.

## 1. Introduction

In the last decades, studies on the impact of (poly)phenols in health and cellular metabolism have accumulated evidence that (poly)phenols are beneficial against human diseases. Particularly in the context of diabetic retinopathy (DR), the beneficial effects of resveratrol (RES) on neovascularization and oxidative stress were observed more than a decade ago [1,2].

(Poly)phenols are members of a large family of compounds, chemically characterized by the presence of one or more hydroxyl groups attached to an aromatic ring. Polyphenols’ metabolites, with potential health-promoting effects, have been studied in experimental cell cultures and animal models, as well as human clinical studies [3,4,5,6,7]. However, when trying to correlate the beneficial health effects described for dietary (poly)phenols and their bioavailability in humans, it is often observed that some of these compounds are poorly absorbed and may undergo several metabolic modification steps, including metabolism in the colon by the resident microbiota. Therefore, it is crucial to look for the metabolite’s bioactivity instead of its parent dietary compounds for relevant insights for its potential preventive or therapeutic effect. Previous studies have shown the presence in human plasma of sulfated metabolites of pyrogallol in volunteers after ingestion of a fruit purée [6], which was found to reach concentrations up to 20 µM. Among the metabolites found in this study, concentrations of pyrogallol-*O*-sulfate (Pyr-s) were found to be significantly higher when compared to the baseline [6], raising some interest in their therapeutic value. 

There are several studies on the anti-diabetic effects of dietary (poly)phenols. These compounds influence glycemia through mechanisms such as the inhibition of glucose absorption in the gut or in peripheral tissues [8]. However, studies specifically related to the effect of (poly)phenols or their metabolites on retinal diseases are scarce. Recently, the beneficial effect of different carotenoids and (poly)phenols in the prevention and treatment of age-related ocular disorders was reviewed, highlighting the role of these plant derivatives in reducing oxidative stress and inflammation [9]. Ola et al. reviewed evidence on the potential metabolic sources and pathways related to the increase of oxidative stress in DR and the role of dietary flavonoids, particularly flavanones, flavanols, isoflavones, flavones, and anthocyanins, in the modulation of redox homeostasis in the diabetic retina [10]. Specifically, epigalloccatechin-3-gallate, the main (poly)phenol component of green tea (leaves of *Camellia sinensis*), was identified as an inhibitor of ocular angiogenesis with influence in ocular vascular permeability after orally administrated in male BALB/c mice [9]. Therefore, oral treatment with nutraceuticals at early stages of DR may represent a reasonable alternative to act upstream of the disease, preventing its progression. The effect of pyrogallol-*O*-sulfate was particularly investigated towards neuroinflammation [3] and cardiomyocyte stress [5]. Moreover this metabolite have the ability to cross of the blood–brain barrier and present neuroprotective properties [3]. As far as we know, no studies were published highlighting the effect of pyrogallol-*O*-sulfate metabolite in ocular structures. 

Vascular endothelial growth factor (VEGF), particularly VEGF-A, is a protein from a large family of angiogenic proteins, that is secreted by different cells in retina, mostly by the basolateral side of RPE. The main functions of these proteins are the regulation of angiogenesis and vascular permeability, with several studies correlating the imbalance in its expression and of its receptors with the severity of DR and age-related macular degeneration (AMD) [11,12]. On the other side, there is one of the most relevant molecules of the RPE secretome, pigment epithelium-derived factor (PEDF), an apically secreted protein from the serine protease inhibitor (serpin) super family. In 1999, PEDF was identified as an anti-angiogenic factor which prompted it immediately being considered as a potential therapeutic target for diabetes mellitus [13]. Shortly after its discovery, low levels of PEDF were correlated with retinitis pigmentosa and Lebers’ congenital amaurosis [13], and also found to be decreased in the retinas from patients with proliferative DR and retinopathy of prematurity [14,15]. The transport of glucose in the human retina was investigated at various ages and found to be exclusively mediated by one of the 12 isoforms of the sodium-independent glucose transporter (GLUT), GLUT-1, expressed in the endothelia of the retinal capillaries of the retina and RPE basal membrane, both part of the blood-retinal barrier (BRB) [16]. Based on a gradient concentration, GLUT-1 adapts the bidirectional transport of glucose to different situations, such as hypoxia, growth factors, and glucose levels itself [17]. 

In this work, we aimed to study the effect of pyrogallol-*O*-sulfate in RPE cells and in the diabetic retina, particularly in the expression of molecules involved in DR-related neovascularization and retinal glial activation with the goal of assessing its potential in ameliorating diabetic retinopathy.

## 2. Results

### 2.1. Metabolic Activity of RPE Cells Is Not Affected by Treatment with Pyrogallol-O-Sulfate

As a first approach, to determine the effect of Pyr-s on retinal cells, we investigated the metabolic activity of RPE cells upon treatment with this metabolite. Although no studies were conducted, so far, aiming to evaluate the ability of these metabolites to cross the BRB, the mean concentration of metabolites found in the plasma of human volunteers 4 h after the ingestion of the fruit purée—6.5 µM, [6]- was chosen to perform the assays. To model the conditions found in the DR retina, we established an in vitro model of hypoxia and hyperglycemia, as described in the Materials and Methods (Section 4.2). To measure the metabolic activity in RPE cells exposed to Pyr-s for 24 h upon exposure to hypoxia for 16 h, an MTT assay was performed.

**Figure 1:**
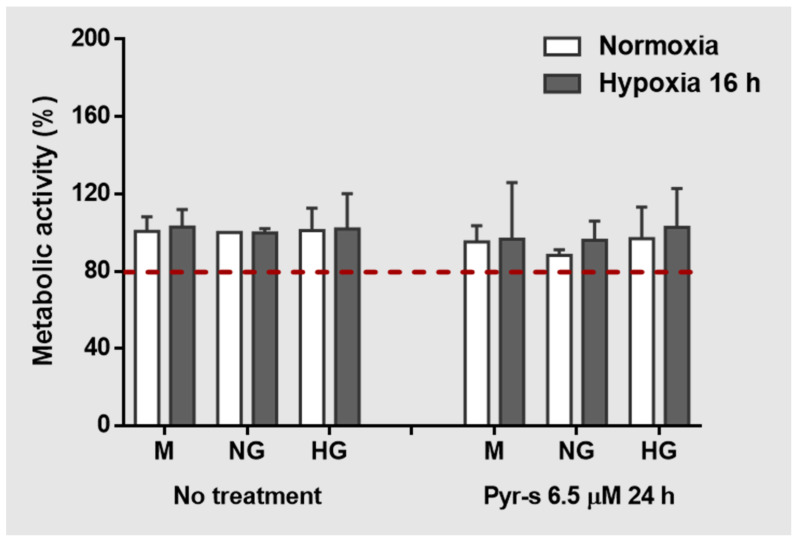
Effect of pyrogallol-*O*-sulfate (6.5 µM, 24 h) on the metabolic activity of RPE cells under high glucose and hypoxia. Viability of RPE cells under normal (NG, 5.5 mM glucose) or high glucose (HG, 25 mM glucose) with chemically induced hypoxia for 16 h (DFO 100 µM), assessed by the MTT assay. *n* = 4. Values are expressed as percentage of control (NG, normal glucose under normoxia). Mannitol (M) was used as the osmolarity control.

As observed in Figure 1, the RPE cells under hypoxia for 16 h were not metabolically affected by the treatment with Pyr-s, with percentages above 80% in all experimental conditions, which is considered the acceptable threshold for most cells in culture [11]. No differences were observed regardless the levels of glucose or treatment.

### 2.2. Pre-Treatment of RPE Cells with Pyrogallol-O-Sulfate before Hypoxia Has No Significant Effect on the Expression of Neovascularization-Related Proteins

(Poly)phenol-derived metabolites have been studied for their potential to prevent neuroinflammation and recent studies show that pre-incubation of microglial cells with pyrogallol-*O*-sulfate before lipopolysaccharide (LPS) insult is able to decrease the expression of several markers of inflammation [3]. Here, we were interested in applying the same hypothesis regarding hypoxia, which has a well-known role in trigger neovascularization. Moreover, the imbalance between pro-and anti-angiogenic proteins is also considered as one of the main contributors to disease progression [12], also promoting neovascularization. However, the new blood vessels are usually weak, fragile, and leaky, which allows proteins, fluids, and debris to enter the retina [13,14].

Our hypothesis is that in RPE cells under high glucose, treatment with Pyr-s before inducing hypoxia prevents the dysregulation of anti-angiogenic PEDF and pro-angiogenic VEGF. Additionally, the expression of the glucose transporter GLUT-1, known to be increased in cells under high glucose and hypoxia [15], is also expected to be downregulated by treatment with Pyr-s before the hypoxic insult.

**Figure 2:**
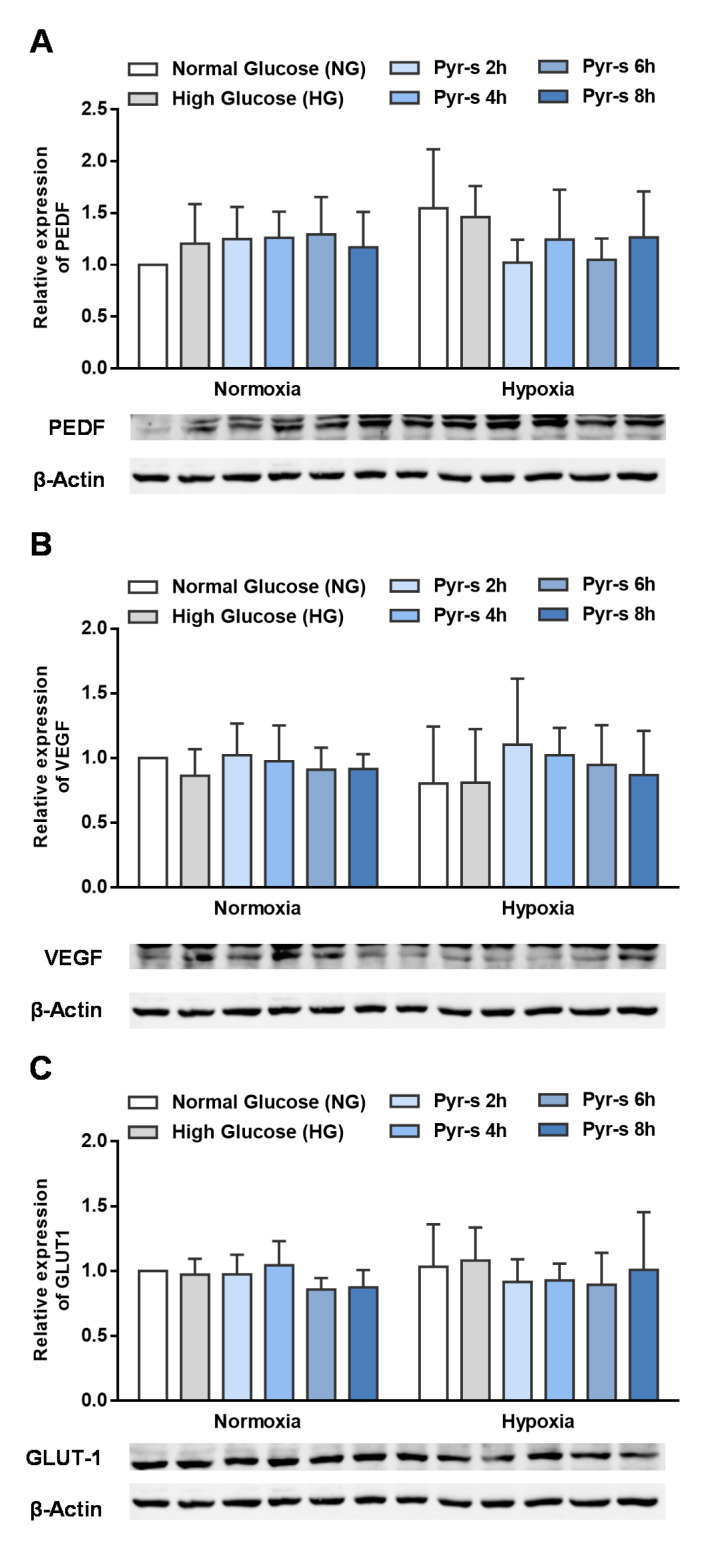
Effect of pre-treatment of pyrogallol-*O*-sulfate on RPE cells for 8 h in normal (5.5 mM glucose) and high (25 mM glucose) glucose before chemically induced hypoxia for 16 h (DFO, 100 µM). RPE cells were exposed to a single dose of Pyr-s, as pre-treatment, up to 8 h before hypoxia. The metabolite was removed from the cell culture after the incubation period. Western blots are a representative image used for the densiometric analysis of (**A**) PEDF, (**B**) VEGF, and (**C**) GLUT-1 protein levels in RPE cells after Pyr-s pre-treatment, normalized by the intensity of β-Actin. *n* = 5.

Figure 2 shows the relative expression of PEDF, VEGF, and GLUT-1 in cells cultured in normal glucose (NG) and exposed to high glucose (HG) for 24 h before treatment under normoxia and hypoxia, with and without Pyr-s treatment. RPE cells were exposed to Pyr-s up to 8 h before hypoxia, with a 2 h interval. Cells in normal glucose and normoxia were considered the control condition, as representative of the physiological situation.

The expression of PEDF (Figure 2A), VEGF (Figure 2B), or GLUT-1 (Figure 2C) is not significantly altered by pre-treatment with Pyr-s before the hypoxic insult, in any of the timepoints evaluated, compared to the physiologic condition (cells under nomoglucose (NG) and normoxia). However, a tendency for pre-treatment of RPE cells with Pyr-s for 2 (*p* = 0.0885) or 6 h (*p* = 0.1148) before hypoxia to decrease the expression of PEDF is observed (Figure 2A), when treated cells under hypoxia are compared to cells under normoglucose and hypoxia (determined by Two-way ANOVA with Dunnett’s multiple comparisons test), as we have previously defined a trend to be considered when the *p* value is below 0.25. Moreover, the expression of the proteins of interest in non-treated RPE cells are not altered by hypoxia or high glucose.

### 2.3. Treatment of RPE Cells with Pyrogallol-O-Sulfate after Hypoxia Does Not Compromise the Expression of PEDF, VEGF, Or GLUT1

Most DR cases are diagnosed at later stages, where ischemia is already established and is the main cause of neovascularization promoted by the increase in the levels of VEGF in the retina. Here we address the hypothesis that RPE cells benefit from treatment with pyrogallol-*O*-sulfate after being exposed to high glucose and chemically induced hypoxia, decreasing the expression of pro-angiogenic VEGF and glucose transporter GLUT-1 and increasing the expression of anti-angiogenic PEDF, therefore restoring the balance in the expression of these proteins. The expression of pro- (VEGF) and anti-angiogenic (PEDF) factors and GLUT-1 was evaluated in RPE cells treated with pyrogallol-*O*-sulfate for 8 h after induction of hypoxia.

**Figure 3:**
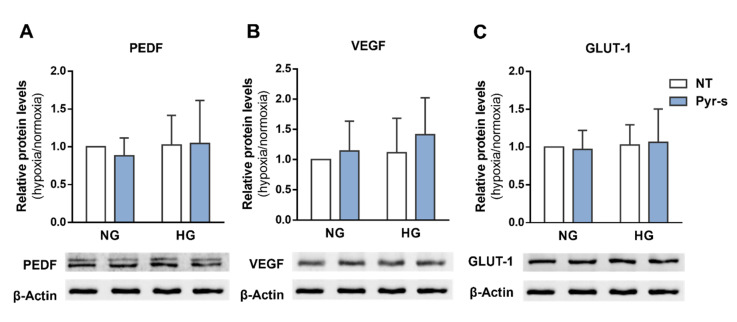
Effect of treatment of RPE cells under normal (NG, 5.5 mM glucose) or high glucose (HG, 25 mM glucose) with pyrogallol-*O*-sulfate for 8 h after chemically induced hypoxia for 16 h (DFO, 100 µM). RPE cells were exposed to a single dose of Pyr-s after induction of hypoxia. Cells were exposed to the metabolite for 8 h. Western blots are a representative image used for the densiometric analysis of (**A**) PEDF, (**B**) VEGF, and (**C**) GLUT-1 protein levels in RPE cells after Pyr-s treatment, normalized by the intensity of β-Actin. *n* = 5. Two-way ANOVA with Sidak’s multiple comparisons test. NT, non-treated; Pyr-s, pyrogallol-*O*-sulfate.

Figure 3 shows the relative expression of PEDF, VEGF, and GLUT-1 in cells without (NT) or with Pyr-s treatment. Values measured in cells under hypoxia were normalized for the respective control in normoxia (Supplementary Figure S5). Cells in normal glucose without treatment were considered the control experimental condition for statistical analysis purposes, as representative of the physiological situation. 

Treatment of RPE cells under high glucose (HG) with Pyr-s for 8 h after hypoxia does not alter the expression of PEDF (Figure 3A), VEGF (Figure 3B), or GLUT-1 (Figure 3C), compared to non-treated cells under normoglucose. When comparing RPE cells treated with Pyr-s with non-treated (NT) cells under high glucose (HG), there is also no differences in the expression of PEDF, VEGF, or GLUT-1.

### 2.4. Treatment of RPE Cells with Pyrogallol-O-Sulfate for 24 H Decreases the Expression of Pro-Angiogenic VEGF

In previous experimental approaches, we analyzed the effect of Pyr-s as protector of the deleterious effects induced by hypoxia on RPE cells and its potential role in reverting those effects after hypoxia is already established. However, we were also interested in the hypothesis of a beneficial effect of more prolonged exposure of RPE cells to Pyr-s. To test this hypothesis, Pyr-s was added to the cell medium 8 h before induction of hypoxia and maintained up to 24 h. The expression levels of PEDF, VEGF, and GLUT-1 were measured in whole cell lysates by Western blot.

**Figure 4:**
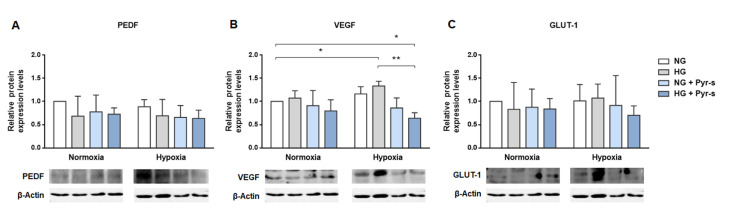
Effect of treatment of RPE cells under normal (NG, 5.5 mM glucose) or high glucose (HG, 25 mM glucose) with pyrogallol-*O*-sulfate for 24 h with chemically induced hypoxia for 16 h (DFO, 100 µM). RPE cells were exposed to a single dose of Pyr-s, 24 h after the increase of glucose in the culture medium, prior to induction of hypoxia. The metabolite was left for 24 h in contact with the cell culture. Western blots are representative images used for the densiometric analysis of (**A**) PEDF, (**B**) VEGF, and (**C**) GLUT-1 protein levels in RPE cells after Pyr-s treatment, normalized by the intensity of β-Actin. *n* = 4. * *p* < 0.05, ** *p* < 0.01 by two-way ANOVA with Dunnett’s multiple comparisons test.

Figure 4 shows the relative expression of PEDF, VEGF, and GLUT-1 in cells in normal glucose (NG) and high glucose (HG) under normoxia and hypoxia with and without treatment with Pyr-s up to 24 h. Cells in normal glucose under normoxia were considered the control experimental condition.

When cells were exposed to Pyr-s before induction of hypoxia, with Pyr-s remaining in the cell medium for 24 h, the expression of VEGF significantly decreased, compared to cells in normal glucose cells and normoxia (Figure 4B). Moreover, cells in high glucose and hypoxia showed an increase in VEGF, as described before, which was prevented by treatment with Pyr-s for 24 h (Figure 4B). The expression of either PEDF or GLUT-1 was not altered by treatment with Pyr-s in these conditions (Figure 4A,C).

Taking together the results from the three experimental approaches, described and schematized in detail in Materials and Methods (Section 4.3), regarding the effect of Pyr-s on the expression of PEDF, VEGF, and GLUT-1 in RPE cells, we can observe that the time of exposure caused different outcomes.

When cells were pre-treated with Pyr-s up to 8 h before being exposed to hypoxia, a tendency to decrease the expression of PEDF (Figure 2A) at different timepoints of treatment was observed. When cells were exposed to Pyr-s after hypoxia was fully established, the expression of our proteins of interest was not altered. The prolonged exposure of RPE cells under high glucose and hypoxia to Pyr-s significantly decreased the expression of pro-angiogenic VEGF, compared to both physiological conditions (normoglycemia and normoxia) and non-treated cells under high glucose and hypoxia (Figure 4B).

To extend our studies to a diabetic animal model, a proof-of-concept protocol was designed based on the least invasive strategy and best approach for a therapeutic effect using the pyrogallol-*O*-sulfate.

### 2.5. A Single Intraocular Injection of Pyrogallol-O-Sulfate Decreases the Expression of Microglial Marker Iba1 in The Retina of a Diabetic Mice

Chronic inflammation has been associated with DR progression, characterized by increased cytokine production and the activation of micro and macroglia cells [16,17]. Although RPE is not the major contributor of cytokines and other inflammatory players, in response to the inflammatory environment, retinal microglia may become overactivated, up-regulating pro-inflammatory factors that induce oxidative stress, neuronal degeneration, and neovascularization [17,18,19,20], affecting other retinal cell layers and promoting disease progression.

We have previously characterized the expression of several markers of DR progression in a spontaneous Type 1 Diabetes *Mellitus* model, the Ins2^Akita^, and found an increase in the expression of microglia marker Iba1 in the neural retina of 6 month-old diabetic mice [12]. At the same time point, the expression of GFAP decreases in diabetic retina, compared to the non-diabetic retina [12]. 

In this context, we analyzed the protein levels of Iba1, as a marker of microglia activation, and GFAP, a glial intermediate filament expressed by astrocytes, in the retina of 4-, 6-, 8-, and 9-month-old male heterozygote Ins2^Akita^ mice two weeks after a single intraocular injection of Pyr-s, targeting the neural retina or the RPE. 

The experimental design is described and schematized in detail in the Materials and Methods (Section 4.7.2). Briefly, diabetic and age-matched non-diabetic animals were randomly divided into two groups, one receiving a single intraocular injection of Pyr-s and the other receiving the vehicle solution. After two weeks, the retina and RPE of the mice were isolated for Western blot analysis of the expression of proteins of interest. The time-points chosen were related to early DR progression in this mice model, where inflammation has a very important role [12,21]. 

**Figure 5:**
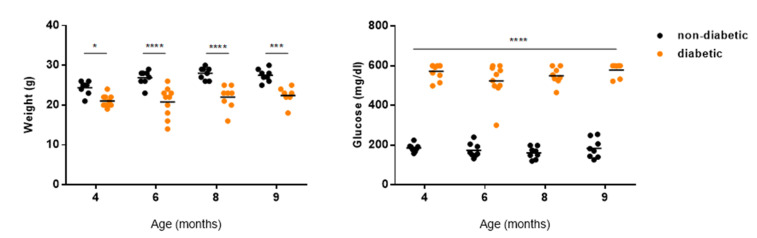
Body weight and blood glucose levels of non-diabetic and diabetic mice of 4, 6, 8, and 9 months of age. Values are represented as mean ± SD. * *p* < 0.05, *** *p* < 0.001, **** *p* < 0.0001 is significantly different compared to non-diabetic mice, determined by Sidak’s multiple comparisons test. *n* = 8 for non-diabetic and *n* = 10 for diabetic.

Blood glucose levels and body weight were measured at the time of injection (Figure 5). Ins2^Akita^ mice weighed significantly less in all time points evaluated compared with control mice (Figure 5). Additionally, Ins2^Akita^ mice displayed significantly increased concentrations of blood glucose compared with non-diabetic mice at all analyzed time points (Figure 5). These data are consistent with previous reports in the literature [21,22].

**Figure 6:**
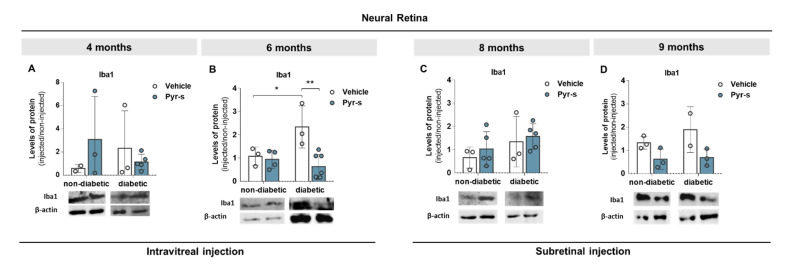
Expression of Iba1 in Ins2^Akita^ mice after a single intravitreal or subretinal injection of pyrogallol-*O*-sulfate. Protein expression levels and representative Western blot images for the densitometric analysis in the retina of Ins2^Akita^ (diabetic) mice and age-matched non-diabetic mice, of 4 (**A**), 6 (**B**), 8 (**C**), and 9 (**D**) months old, intravitreal or subretinally injected with vehicle or Pyr-s. Protein levels were normalized to β-actin. Data are expressed as mean ± SD (*n* = 2 to 6 mice/group), * *p* < 0.05, ** *p* < 0.01 by two-way ANOVA (age and genotype) with Sidak’s multiple comparison test.

**Figure 7:**
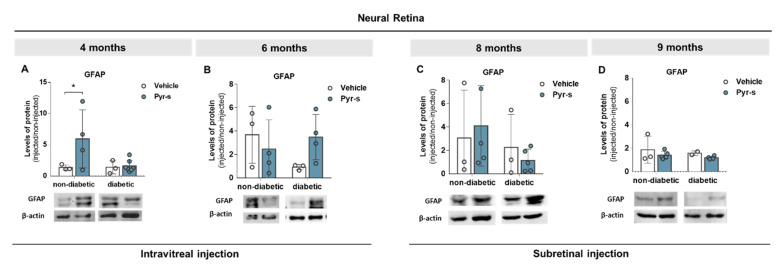
Expression of GFAP in Ins2^Akita^ mice after a single intravitreal or subretinal injection of pyrogallol-*O*-sulfate. Protein expression levels and representative Western blot images for the densitometric analysis in the retina of Ins2^Akita^ (diabetic) mice and age-matched non-diabetic mice, of 4 (**A**), 6 (**B**), 8 (**C**), and 9 (**D**) months old, intravitreal or subretinally injected with vehicle or Pyr-s. Protein levels were normalized to β-actin. Data are expressed as mean ± SD (*n* = 2 to 6 mice/group) * *p* < 0.05 by two-way ANOVA (genotype and treatment) with Sidak’s multiple comparison test.

The results for the expression of Iba1 and GFAP, normalized for levels of expression of the contralateral non-injected eye, are plotted in Figure 6 and Figure 7, respectively. No differences were observed in the expression of any of the proteins evaluated upon intraocular injection of vehicle solution, when the vehicle was compared to the contralateral non-injected eye, with an exception for the expression of GFAP in non-diabetic retina of 6-month-old animals (Supplementary Figures S1–S4).

Our results show that the expression of Iba1 was not altered by intraocular injection of either vehicle or Pyr-s in 4-month-old animals (Figure 6A). However, as previously observed [12], the expression of Iba1 increased in 6-month-old mice when comparing the retinas injected with the PBS vehicle from non-diabetic and diabetic mice (Figure 6B). The treatment of diabetic retinas with the (poly)phenol-derived metabolite Pyr-s effectively decreased the expression of Iba1 (Figure 6B). No differences were observed in the expression of Iba1 in mice subretinally injected at 8 months (Figure 6C) or 9 months old (Figure 6D).

Regarding the GFAP, an increase in its expression was observed in 4-month-old non-diabetic mice upon intravitreal injection of Pyr-s (Figure 7A). Moreover, as previously described by our group [12], a decrease in the expression of GFAP was observed in 6 month-old mice when non-diabetic retinas were compared to diabetic retinas (Figure 7B). Treatment of diabetic retinas with Pyr-s tends to increase the expression of GFAP (*p* = 0.2468) at the same time-point (Figure 7B). No differences in the expression of GFAP were observed in older mice, subretinally injected, although there was a tendency of treatment with Pyr-s decreasing the expression of GFAP (*p* = 0.2490), when non-diabetic retinas of 8-month-old mice were compared to diabetic (Figure 7C).

In a previous study, we showed that the expression of Iba1 tends to increase in the 9-month-old diabetic retina but is not altered in older mice [12]. Here, we analyzed the expression of Iba1 and GFAP in 8- and 9-month-old mice to understand if the administration of Pyr-s before an advanced stage of DR (as previously established to be at 9 months) has a positive effect on retinal homeostasis. Treatment of diabetic 9-month-old mice with a single subretinal injection tends to decrease the expression of Iba1 in the neural retina (Figure 6D) and has no effect in the 8-month-old mice (Figure 6C). Furthermore, the expression of GFAP was not affected by Pyr-s in 8- (Figure 7C) or 9-month-old (Figure 7D) animals.

### 2.6. A Single Subretinal Injection of Pyrogallol-O-Sulfate Decreases the Expression of Pro-Angiogenic VEGF in the RPE of a Diabetic Mouse Model

We have previously documented the dysregulation of the balance between pro- and anti-angiogenic factors in the Ins2^Akita^ mouse, with a significant increase in the expression of VEGF with a concomitant decrease in the expression of PEDF in the neural retina of 9-month-old mice [12].

**Figure 8:**
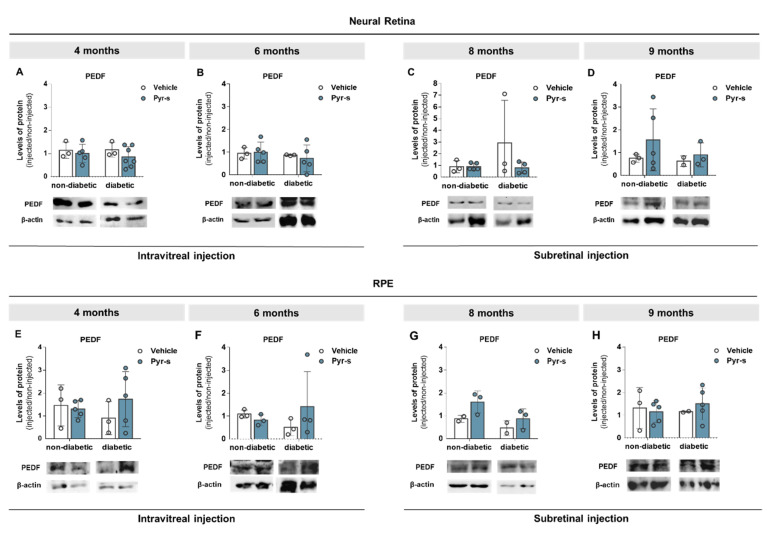
Expression of PEDF in Ins2^Akita^ mice after a single intravitreal or subretinal injection of pyrogallol-*O*-sulfate. Protein expression levels and representative Western blot images for the densitometric analysis in the retina (**A**–**D**) and RPE (**E**–**H**) of Ins2^Akita^ (diabetic) mice and age-matched non-diabetic mice, 4- (**A**–**E**), 6- (**B**–**F**), 8- (**C**–**G**), and 9- (**D**–**H**) months old, injected intravitreally or subretinally with vehicle or Pyr-s. Protein levels were normalized to β-actin. Data are expressed as mean ± SD (*n* = 2 to 5 mice/group). Two-way ANOVA (genotype and treatment) with Sidak’s multiple comparison test.

**Figure 9:**
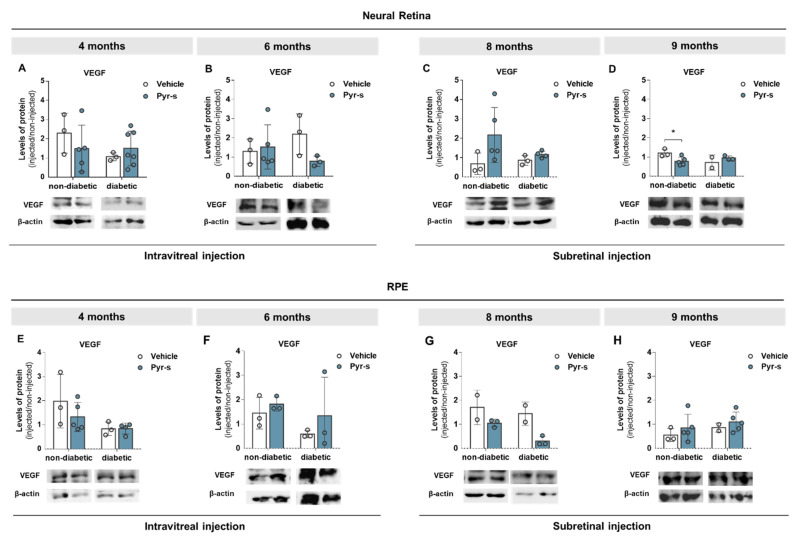
Expression of VEGF in Ins2^Akita^ mice after a single intravitreal or subretinal injection of pyrogallol-*O*-sulfate. Protein expression levels and representative Western blot images for the densitometric analysis in the retina (**A**–**D**) and RPE (**E**–**H**) of Ins2^Akita^ (diabetic) mice and age-matched non-diabetic mice, of 4 (**A**–**E**), 6 (**B**–**F**), 8 (**C**–**G**), and 9 (**D**–**H**) months old, injected via intravitreal or subretinal route with vehicle or Pyr-s. Protein levels were normalized to β-actin. Data are expressed as mean ± SD (*n* = 2 to 5 mice/group). * *p* < 0.05 by Two-way ANOVA (genotype and treatment) with Sidak’s multiple comparison test.

**Figure 10:**
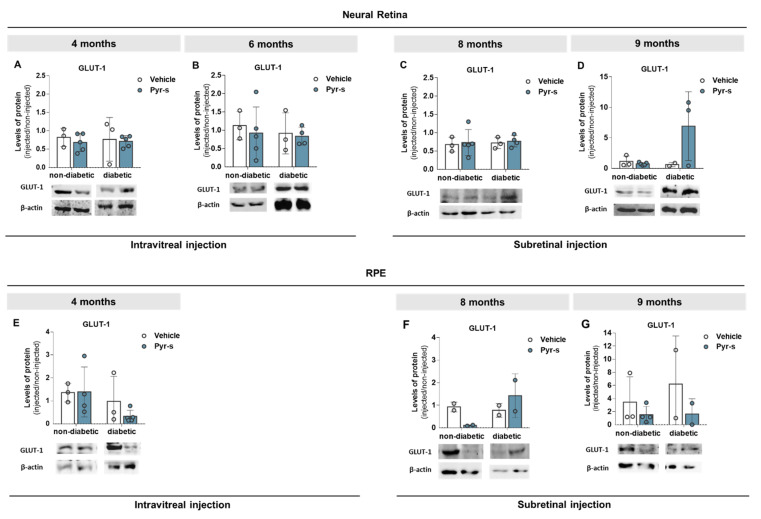
Expression of GLUT-1 in Ins2^Akita^ mice after a single intravitreal or subretinal injection of pyrogallol-*O*-sulfate. Protein expression levels and representative Western blot images for the densitometric analysis in the retina (**A**–**D**) and RPE (**E**–**G**) of Ins2^Akita^ (diabetic) mice and age-matched non-diabetic mice, of 4 (**A**,**E**), 6 (**B**), 8 (**C**,**F**), and 9 (**D**,**G**) months old, injected via intravitreal or subretinal route with vehicle or Pyr-s. Protein levels were normalized to β-actin. Data are expressed as mean ± SD (*n* = 2 to 5 mice/group). Two-way ANOVA (age and genotype) with Sidak’s multiple comparison test.

To further evaluate the effect of pyrogallol-*O*-sulfate in the expression profile of pro- and anti-angiogenic factors with disease progression in the Ins2^Akita^ mouse, we evaluated the protein levels of PEDF (Figure 8) and VEGF (Figure 9) in neural retina and RPE cells of 4-, 6-, 8-, and 9-month-old mice two weeks after intraocular injection of Pyr-s, as described in Materials and Methods (Section 4.7.2). We were also interested in evaluating the expression of glucose transporter, GLUT-1, as its expression was previously reported to be imbalanced in diabetic retinas [15] (Figure 10). 

The expression of anti-angiogenic PEDF was not altered in the neural retina of either non-diabetic or diabetic mice, either by intraocular injection of vehicle or Pyr-s, at any of the time-points evaluated (Figure 8A–D). Similarly, no differences were observed in the RPE, independently of the genotype (Figure 8E–H).

The expression of pro-angiogenic VEGF was also evaluated. When analyzing the effect of a single intravitreal injection of Pyr-s in animals of 4 months of age, the expression of VEGF was not altered (Figure 9A); however, upon treatment with Pyr-s, the expression of VEGF tended to decrease (*p* = 0.1761) in the diabetic retina of 6-month-old mice (Figure 9B), compared to vehicle. Unexpectedly, the expression of VEGF tended to increase (*p* = 0.086) in non-diabetic retinas with 8-month-olds, upon treatment with Pyr-s (Figure 9C), but effectively decreased later in animals of 9 months (Figure 9D), with no alterations in the expression of this protein in older diabetic retinas. Regarding the RPE, treatment with Pyr-s did not impact the expression of VEGF in younger mice (Figure 9E,F), nor in older mice (Figure 9G,H), despite a tendency to decrease VEGF in diabetic mice (Figure 9G) which needs further confirmation.

A tendency to increase the expression of VEGF was previously reported by our group in the RPE of diabetic Ins2^Akita^ mice at 9 months of age [12]. Although Pyr-s treatment did not have an effect in the expression of VEGF in the RPE of diabetic animals of 9 months of age, it also did not increase its expression, revealing that it is not a promoter of the imbalance in the expression of proteins involved in neovascularization. 

Similarly to what was observed for PEDF and VEGF, the expression of GLUT-1 was not significantly altered by intraocular treatment with Pyr-s. In general, the expression of GLUT-1 in the neural retina of both non-diabetic and diabetic mice remained unchanged at all time-points, when compared to the injection vehicle (Figure 10D). Regarding the expression levels at RPE, a tendency to decrease (*p* = 0.1255) the expression of glucose transport in diabetic RPE of 4-month-old mice compared to non-diabetic mice (Figure 10E) was observed. We were unable to detect the expression of GLUT-1 in samples from 6-month-old mice, which can most probably be attributed to the low levels in RPE protein isolates. No differences were detected in the RPE of older mice subretinally injected with Pyr-s (Figure 10F,G).

## 3. Discussion

Classically regarded as a progressive microvascular complication of diabetes with an inflammatory background, DR remains one of the major causes of acquired visual impairment and/or blindness among the diabetic population [13,23,24]. Although the exact mechanism underlying DR pathophysiology is not yet fully understood, it is believed that the chronic exposure to hyperglycemia triggers a cascade of biochemical and physiological changes, causing inner BRB breakdown [25,26]. These events are potentiated by oxidative stress and inflammation, which promote increase in the production of reactive oxidative species (ROS), cytokines, and growth factors, altogether contributing to visual impairment or irreversible blindness [13].

As mentioned before, one of the major characteristics of DR is vascular changes along the progression of the disease, trigged by chronic and sustained hyperglycemia and hypoxia. Due to retinal ischemia during diabetes, the balance between pro- and anti-angiogenic molecules is disrupted, leading to up-regulation of pro-angiogenic VEGF and down-regulation of anti-angiogenic PEDF [27,28]. Our group has shown that sustained expression of PEDF up to 3 months by an episomal vector, both in cultures of human RPE cells and in the retina of a DR mice model after a single subretinal injection, decreased VEGF, GLUT1, and inflammation markers. As a consequence of the imbalance mostly between these two growth factors, neovascularization is promoted but the new blood vessels are usually weak, fragile, and leaky, which allows proteins, fluids, and debris to enter the retina [13,14]. Moreover, we have shown that in a DR cell model, hyperglycemia and hypoxia directly affect GLUT-1 expression and the secretory function of RPE cell [15]. It was found that not only the expression of GLUT-1 is increased when RPE cells are in hypoxia under high glucose conditions, but also its membrane fraction increases under the same conditions. The consumption of glucose is also affected, pointing to an increase in the GLUT-1 expression to achieve an effective glucose transport [15].

At present, there are few effective treatments for DR. Tight glycemic control is the most efficient therapy to slow the progression of DR [29,30]. Blood pressure should also be closely monitored, since hypertension aggravates the disease by increasing blood flow and causing mechanical damage to the vascular endothelial cells, thus stimulating the production of VEGF [13,14]. However, current treatments for DR are designed to act on later stages of this pathology, targeting ocular lesions resulting from the disease progression. In pathologies associated with neovascularization, such as DR or wet AMD, intravitreal administration of monoclonal antibodies or fusion proteins that block VEGF activity is considered as first line therapeutic approach [31]. Considering the growing numbers of diabetes incidence and prevalence [32,33] and that these forms of treatment only avoid the progression of DR temporarily, there is a need to seek therapeutic alternatives that either have longer lasting effects or can target DR in earlier stages of progression.

Knowing that targeting DR remains a challenge, it is important to understand how we can prevent its progression; thus, our main goal is to investigate different and novel therapeutic approaches towards DR, based on the biological proprieties of (poly)phenol-derived metabolites.

There are several studies on the anti-diabetic effects of (poly)phenols showing these compounds to influence the glycemia status through different mechanisms such as the inhibition of glucose absorption in the gut or in peripheral tissues [8]. Some studies show the inhibition of glucose transporters by (poly)phenols. For example, onion (poly)phenols have the capacity to protect diabetic patients from oxidative stress and (poly)phenols from vegetables act as potent anti-diabetic agents by decreasing the levels of blood glucose and increasing plasma insulin [34,35].

Studies specifically related to the effect of (poly)phenols or their metabolites on retinal diseases are scarce. However, the interest in this topic increased in the last decade, particularly in studies on the effect of resveratrol, as reviewed by Popescu et al. [36]. More recently, the beneficial effect of different carotenoids and (poly)phenols in the prevention and treatment of age-related ocular disorders was reviewed, highlighting the role of these compounds in oxidative stress and inflammation [9]. In particular, in vitro and in vivo studies have revealed that a variety of nutraceuticals have significant antioxidant and anti-inflammatory properties that may inhibit the early diabetes-driven molecular mechanisms that induce DR, reducing both the neural and vascular damage typical of DR [37,38].

The presence of sulfated metabolites of pyrogallol was confirmed for the first time by Santos and colleagues in human plasma from volunteers after ingestion of a fruit purée [6]. The metabolite Pyr-s was found in in some volunteers, in concentrations up to 20 µM. Pyr-s metabolites have been recently studied in different models of neurodegenerative and cardiovascular disorders, showing the ability of these small molecules to cross the blood-brain barrier, modulate the brain cell towards neuroprotection, and affect cardiomyocyte beating following prolonged stimulation [3,5]. Furthermore, it was shown for the first time that Pyr-s was also effective in preventing oxidative damage in brain endothelial cells and attenuating inflammation in microglial cells [3]. 

Here we aim to address the effect of Pyr-s in the expression of proteins involved in inflammation and neovascularization, using RPE cells and a diabetic mouse model. To the best of our knowledge, this is the first time this (poly)phenol-derived metabolites have been tested in RPE cells, so as a first approach, it was important to evaluate how RPE cells are affected by the presence of Pyr-s. RPE cells, under high glucose and hypoxia, were not metabolically affected by treatment with Pyr-s (6.5 µM, 24 h).

The levels of PEDF and VEGF, whose expression is imbalanced in DR patients, as well as the glucose transporter GLUT-1 were analyzed in RPE cells treated with a single dose of Pyr-s in three different protocols. When Pyr-s was added before the induction of hypoxia, the expression of anti-angiogenic PEDF tended to decrease at certain timepoints. On the other hand, no alterations in the expression of the proteins evaluated were observed when Pyr-s was added after the establishment of hypoxia. However, the expression of VEGF was effectively decreased when RPE cells were exposed to Pyr-s for 24 h, starting before the induction of hypoxia. 

This compound was described as being further metabolized by brain endothelial cells [3]. Considering this, the same characterization in RPE cells upon exposure to Pyr-s could help understand whether these metabolites are further metabolized by the cell, to further study their physiological relevance, and possibly explain the differences in the expression of the proteins analyzed according to the time of exposure to Pyr-s. Moreover, neither PEDF nor VEGF secreted levels were analyzed in this study. Secreted proteins’ levels should be measured in the cell culture medium to better understand if treatment with Pyr-s has an impact in the secretory function of RPE cells.

Several studies correlate the bioactivity of (poly)phenol-derived metabolites with an improvement in vascular function in healthy humans, linked to the presence of newly identified plasma metabolites [39,40]. In addition, a study showed that epigalloccatechin-3-gallate, the main plant-derived (poly)phenol of green tea, effectively protected RPE cells from cell death and attenuated mRNA expressions of key angiogenic factors (MMP-9, VEGF, VEGFR2) by inhibiting the generation of ROS, with further evidence on the inhibition of ocular neovascularization and vascular permeability [9]. In the same line, resveratrol, also a plant-derived (poly)phenol, present in red wine, as well as other dietary (poly)phenols, were shown to have anti-angiogenic properties, as reviewed in [41]. Our results point to a potential anti-angiogenic effect of Pyr-s on RPE cells that is dependent on the time of exposure of the cells to the bioactive metabolite. In addition, further studies focused on the vascular features performed in a more robust hyperglycemia retinal model would help to understand the extension of the potential of treatment with Pyr-s in DR prevention and progression.

Regarding the levels of GLUT-1, which were not affected by Pyr-s in any of the experimental setups, an evaluation of the glucose consumption by the cells may be useful to determine if Pyr-s affects glucose uptake without significantly changing the expression of GLUT-1 in the cell membrane.

Further studies may be conducted to assess the effect of Pyr-s in RPE cells, either with a role in angiogenesis or ocular inflammation. Assessing the secreted levels of other cytokines such as TNF-α or IL-1β would allow a full characterization of the secretory profile of RPE cell upon treatment with Pyr-s and, therefore, the biological outcome towards inflammation. Moreover, the distinct outcomes in RPE cells upon treatment with Pyr-s highlights the importance of different experimental approaches in studying multifactorial diseases such as DR. 

To further study the therapeutic potential of Pyr-s, we evaluated, after intraocular injection of the metabolite in Ins2^Akita^ mice, the expression of several proteins known to be involved either in the DR characteristic inflammatory process or in neovascularization. Diabetic and age-matched non-diabetic animals were grouped by ages covering different stages of DR progression, and the eye injected with vehicle or Pyr-s at the same concentration as used in vitro. A single injection was performed, intravitreally or subretinally, and the expression of several proteins of interest was measured in the neural retina and RPE. We have previously characterized the expression of several markers of DR progression in a spontaneous Type 1 Diabetes *Mellitus* model, the Ins2Akita, and found an increase in the expression of microglia marker Iba1 in the neural retina of 6-month-old diabetic mice [19]. At the same time point, the expression of GFAP decreases in diabetic retina, compared to the non-diabetic retina [19].

The expression of the microglia marker, Iba1, significantly decreased in diabetic retina after a single intravitreous injection of Pyr-s in 6-month-old animals. In accordance with our results, digested raspberry metabolites used at physiological levels previously exhibited anti-inflammatory activity, not only by the reduction of Iba1 expression, but also by inhibiting the release of nitric oxide and TNF-α [42]. Consistent with the activation of microglia described in both early DR patients and other animal models, our results in the Ins2^Akita^ mouse showed an increased expression of Iba1 [21,23]. This seems to be thwarted by treatment with pyrogallol-*O*-sulfate in animals with early (6-month-old) DR features, highlighting the potential of Pyr-s to prevent microglial activation in the diabetic retina.

In the normal retina, astrocytes label more heavily with anti-GFAP than the Müller cell endfeet (which predominately express the type-III intermediate filament vimentin), but after injury, there is a rapid up-regulation of GFAP by Müller cells [43,44]. In our studies, elevated GFAP expression was observed early upon intravitreal injection of Pyr-s in 4-month-old non-diabetic mice and reached values close to the control in later stages of the disease. A tendency to increase the expression of GFAP in 6-month-old diabetic retinas upon intravitreal injection of Pyr-s was observed. As it is described that macroglia undergoes prominent changes in early DR, which includes a decrease in the astrocytic population and reduced coverage of retinal blood vessels by glial cell processes [43,45], and to date no study has been able to connect increased levels of GFAP to any functional outcome, these results need further confirmation regarding the astrocytic population to evaluate whether Pyr-s has a role in maintaining the number of astrocytes, protecting the retinal blood vessels, or if this tendency to increase the expression of GFAP enhances macroglia activation promoting the progression of DR. Furthermore, an increased permeability of BRB in the early stages of DR in Ins2^Akita^ mice [21,22] has been described, in line with the decreased levels of the astrocyte marker GFAP previously observed in all tested ages [12]. Because the injection of the vehicle does not increase the expression of GFAP, when the diabetic retina is compared to non-diabetic (Supplementary data), our hypothesis relies on a non-pathological role of Pyr-s driven by GFAP expression. 

Concerning the levels of proteins contributing to neovascularization, there is no statistical significance in the results regarding the expression of anti-angiogenic PEDF, but there was a tendency to decrease the expression of pro-angiogenic VEGF in the diabetic retina of the 6-month-old animals upon injection with Pyr-s. In agreement with what is described for patients with proliferative DR, we previously observed an increase of VEGF in late stages of the disease in Ins2^Akita^ mice both in the neural retina and the RPE [12,22]. However, at earlier stages, we observed significantly decreased levels of VEGF in RPE cells and a slight decrease in the neural retina [12]. Here, a single intraocular injection of Pyr-s at the physiological concentration found in human plasma tended to decrease the expression of VEGF at earlier stages. These studies, despite requiring further addressing, together with those reviewed by Cao et al. [41], raise significant interest in (poly)phenols as an important group of therapeutic natural compounds in the treatment of neovascular-dependent disorders.

The expression of glucose transporter, GLUT-1, also tended to decrease in the RPE of 4-month-old mice treated with Pyr-s by intravitreal injection, showing that it is possible to have an effect promoted by Pyr-s in the outer layer of the retina after intravitreal administration. Additionally, the expression of GLUT-1 decreases in non-diabetic RPE from 8-month-old mice upon a single subretinal injection of Pyr-s.

Overall, our results point to a role of pyrogallol-*O*-sulfate in different stages of diabetic retinopathy progression by affecting the expression of pro-angiogenic VEGF in in vitro RPE cells and preventing microglia activation earlier after a single intraocular injection in diabetic mice models. 

## 4. Materials and Methods

### 4.1. Cell Culture

D407 cells, a human spontaneously transformed RPE cell line [46], were kindly provided by Dr. Jean Bennett (University of Pennsylvania, Philadelphia, PA, USA). Cells were maintained in 5% CO_2_–95% air at 37 °C and grown in Dulbecco’s Modified Eagle Medium (DMEM) with 5.5 mM D-glucose (Sigma-Aldrich, St. Louis, MO, USA) supplemented with 1% penicillin/streptomycin (Sigma-Aldrich, St. Louis, MO, USA), 1% glutamine (Sigma-Aldrich, St. Louis, MO, USA), and 5% fetal bovine serum (FBS, Sigma-Aldrich, St. Louis, MO, USA). For sub-culturing, cells were dissociated with a trypsin-Ethylenediaminetetraacetic Acid (EDTA) solution (Sigma-Aldrich, St. Louis, MO, USA), split 1:5 and cultured in 21 cm^2^ culture flasks (Orange Scientific, Braine-lÁlleud, Belgium). Culture medium was changed every 2 days.

### 4.2. Glucose and Hypoxia

For the experiments regarding the effect of glucose, RPE were seeded at a density of 3 × 10^5^ cells/well in a 6-well plate (Orange Scientific, Braine-lÁlleud, Belgium). For each independent experiment, cells were exposed to either DMEM with 5.5 mM glucose, to mimic a physiological concentration, DMEM with 25 mM glucose, to mimic a hyperglycemic condition, or DMEM with 5.5 mM of d-glucose containing d-Mannitol (AppliChem GmbH, Darmstadt, Germany) with a final concentration of 25 mM, as an osmolarity control, as it is known to be metabolically inert in mammalian cells as energy source [47].

Desferrioxamine (DFO, Sigma-Aldrich, St. Louis, MO, USA), an iron chelating agent, was used as a hypoxia-mimetic agent at a final concentration of 100 µM [48,49]. Under normoxic conditions and in the presence of iron, hypoxia-inducible factor-1α is hydroxylated and subsequently degraded by the proteasome. In the presence of DFO, the iron required for the enzymatic activity of prolyl hydroxylases is removed by its chelating capacity, allowing hypoxia-inducible factor-1α to be stabilized and dimerize with its β-subunit, originating a functional complex that is translocated to the nucleus [48].

### 4.3. Treatment with (Poly)Phenol-Derived Metabolites

**Figure 11:**
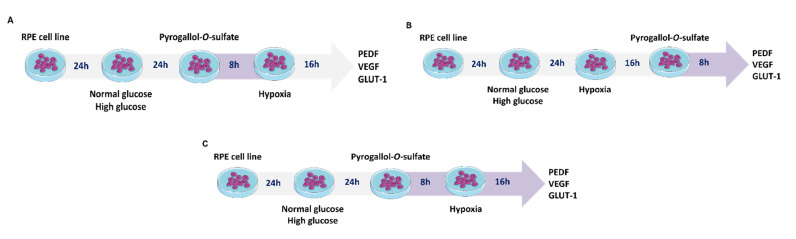
Schematic representation of the different protocols designed to evaluate the effect of pyrogallol-*O*-sulfate in the expression of proteins of interest in RPE cells. Cells were kept in culture in standard conditions under normal glucose levels (5.5 mM) until exposure to high glucose medium (25 mM). (**A**) A single dose as pre-treatment up to 8 h before hypoxia. The metabolite was removed from the cell culture after the incubation period. (**B**) A single dose after induction of hypoxia. Cells were exposed to the metabolite for 8 h. (**C**) A single dose, 24 h after the increase of the glucose in the culture medium, prior to induction of hypoxia. The metabolite was left for 24 h in contact with the cell culture. The expression of PEDF, VEGF, and GLUT-1 was analyzed in whole cells lysates by Western blot.

Pyrogallol-*O*-sulfate was chemically synthesized as described by Pimpão et al [6]. To evaluate the effect of the polyphenol metabolite, RPE cells were plated with DMEM with 5.5 mM glucose to mimic a physiological concentration. The next day, the medium was changed to DMEM with 5.5 mM glucose or DMEM with 25 mM glucose to mimic a hyperglycemic condition. Depending on the therapeutic approach to test, pyrogallol-*O*-sulfate (Pyr-s, 6.5 µM) was incubated in media without FBS in three different protocols represented below (Figure 11). 

### 4.4. Protein Extraction and Quantification

#### 4.4.1. Whole Cell Lysates

To obtain cellular lysates, cells were washed with cold PBS 1X and homogenized in radioimmunoprecipitation assay buffer (RIPA) (50 mM Tris-Hydrochloric acid (HCl, Sigma-Aldrich, St. Louis, MO, USA) pH 7.4, 1% NP-40 (Millipore, Burlington, MA, USA), 0.25% Na-deoxycholate (Sigma-Aldrich, St. Louis, MO, USA), 150 mM Sodium chloride (Sigma-Aldrich, St. Louis, MO, USA) and 1 mM EDTA (Sigma-Aldrich, St. Louis, MO, USA) supplemented with protease inhibitors cocktail 1× (Roche, Basel, Switzerland). The cells were then scrapped from the bottom of the well, kept on ice for 20 min, centrifuged (16.100× *g*, 20 min, 4 °C) and the supernatant collected and stored at −20 °C until further analysis.

#### 4.4.2. Protein Quantification

Protein concentration was determined by the Bradford Method [50], using the Bio-Rad protein assay dye reagent concentrate (BioRad, Hercules, CA, USA) in cellular lysates and retinal tissue. Bovine serum albumin (NZYTech, Lisboa, Portugal or Thermo Fisher Scientific, Waltham, MA, USA) was used as standard (1 mg/mL; 0.5 mg/mL; 0.25 mg/mL; 0.125 mg/mL; and 0.0625 mg/mL) prepared in miliQ water. Samples were also diluted in miliQ water in 1:5 or 1:10 ratios for cell lysates and retinas, respectively. The cell and standard samples were loaded into a 96-well plate, and the dye reagent added to each well. The absorbance was read in a Biotrak II Plate reader (Amersham Biosciences, Amersham, UK) at 595 nm and concentration of protein was determined by linear regression.

### 4.5. MTT Assay

D407 cells were seeded at a density of 2 × 10^4^ cell/well in a 48-well plate, with a volume of DMEM of 500 µL/well with 5.5 mM glucose (supplemented with 1% penicillin/streptomycin and 5% FBS), hence considered the physiological condition. After 24 h, the medium was changed, and cells were exposed to different conditions as previously described in Section 4.2. Treatment with the polyphenolic metabolite pyrogallol-*O*-sulfate was performed according to the protocol described in Figure 11C.

In each well, 25 µL of MTT (Sigma-Aldrich, St. Louis, MO, USA) at a final concentration of 0.25 mg/mL was incubated at 37 °C for 3 h. At the end of the incubation period, the formazan was dissolved with 250 µL of 0.04 N HCl in isopropanol 100% and the absorbance was measured in a Biotrak II Plate reader (Amersham Biosciences, Amersham, UK) at 540/620 nm. 

### 4.6. Immunoblotting

The samples were diluted in RIPA buffer with 4 × Laemmli Sample Buffer (BioRad, Hercules, CA, USA) and heated at 95 °C for 5 min for deoxyribonucleic acid (DNA) denaturation. A total of 30 µg of protein was loaded in a 10 to 15% SDS acrylamide gel, as suitable, along with 3 µL of a PageRuler Plus Pre-stained Protein Ladder 2× (Thermo Fisher Scientific, Waltham, MA, USA). Electrophoresis was performed at 100–120 V in electrophoresis buffer (25 mM Tris-HCl, 192 mM Glycine (Enzymatic, Loures, Portugal) and 0.1% SDS (AppliChem GmbH, Darmstadt, Germany) until the dye reached the bottom of the gel. 

Proteins were electrotransferred to polyvinylidene difluoride (GE Healthcare Life Sciences, Chicago, IL, USA) previously activated for 20 s with methanol, followed by deionized H_2_O for 20 s and cold transfer buffer (0.192 M glycine, 0.025 M Tris pH 8.3, 10% methanol) for 5 min, or nitrocellulose (NC, (GE Healthcare Life Sciences, Chicago, IL, USA)) membrane through a semi-dry transfer method for 20 min and 20 V (Trans-Blot Turbo^®^, BioRad, Hercules, CA, USA). The membranes were then blocked with 5% bovine serum albumin in Tris buffered saline (TBS) with 0.1% Tween-20 (Sigma-Aldrich, St. Louis, MO, USA) (TBS-T) for 1 h at room temperature, with gently agitation. Primary antibodies, listed in Table 1, diluted in 5% bovine albumin serum in TBS-T were incubated overnight at 4 °C with gentle agitation. The membranes were subsequently washed with TBS-T and incubated with the correspondent secondary antibodies IgG conjugated with HRP (Santa Cruz Biotechnology, Dallas, TX, USA) for 1 h at room temperature. After washing three times for 5 min each with TBS-T, the enhanced chemiluminescence Select Western Blotting Detection Reagent (1:1, GE Healthcare Life Sciences, Chicago, IL, USA) was used for visualization in a ChemiDoc^TM^ imaging System (BioRad, Hercules, CA, USA). To ensure that samples were evenly loaded, the membranes were stripped and re-probed for β-actin. 

For stripping, the membrane was washed three times with TBS-T for 5 min, incubated for 5 min with 0.2 N sodium hydroxide (NaOH, Sigma-Aldrich, St. Louis, MO, USA) or mild stripping solution (glycine, SDS, Tween-20 pH = 2.2), followed by a triple TBS-T wash for 5 min and incubated with the new primary antibody.

### 4.7. Animals

Male C57BL/-Ins2^Akita^-6J heterozygote and C57BL/6J age-matched (wild-type) mice from The Jackson Laboratory (Bar Harbor, ME, USA) were used. These mice present a dominant point mutation in the insulin 2 gene that spontaneously induces diabetes, developing hyperglycemia as early as 4 weeks of age [21]. With the progression of hyperglycemia, diabetic mice can develop retinal complications similar to what is observed in DR [21], making them a good model to study early alterations of DR.

#### 4.7.1. Housing

Animals were maintained and handled in accordance with the Portuguese and European Laboratory Animal Science Association (FELASA) Guide for the Care and Use of Laboratory Animals, the European Union Council Directive 2010/63/EU for the use of animals in research and the Association for Research in Vision and Ophthalmology (ARVO) for the use of animals in ophthalmic and vision research. Animal experiments have been performed under the approval of the Portuguese national body (DGAV—Direção Geral de Alimentação e Veterinária) and the institutional body.

Mice were housed in individually ventilated cages, under controlled temperature, with continuous access to food and water, and on a 12 h dark/light cycle. To confirm the diabetic phenotype, blood glucose levels were measured 2 months after birth, with a drop of blood obtained by a tail cut on a reactive glucose strip (Contour Next, Ascensia Diabetes Care, Basel, Switzerland). The mice presenting blood glucose levels equal or higher than 250 mg/dL were considered diabetic [21].

Male diabetic C57BL/-Ins2^Akita^-6J and non-diabetic C57BL/-Ins2^Akita^-6J age-match mice (The Jackson Laboratory, Bar Harbor, ME, USA) were used as DR model. The main goal of the following experimental design was to assess the effect of Pyr-s on the expression of proteins involved in inflammation and neovascularization in the neural retina and RPE of animals at different ages (4, 6, 8, and 9 months of age). These time-points were set taking into consideration previous work where molecular and cellular alterations were observed consistent with clinical observations of DR progression [21]. Moreover, we have previously found alterations in protein levels important for DR pathophysiology in both neural retina and RPE of the Ins2^Akita^ mice up to 12 month old [12]. As such, the following experiments were designed for a role of Pyr-s in both the early stages of DR but also in later stages of the disease. The estimated number of subjects in each group, for a statistically significant difference (*p* < 0.05), with a difference in means of 50%, was at least 5 animals per group. For this study, a total of 70 animals were used.

#### 4.7.2. Intraocular Injections of Pyrogallol-*O*-Sulfate in C57BL/Ins2^Akita^ Mice

**Figure 12:**
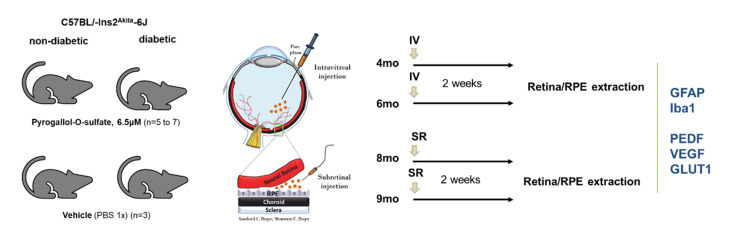
Schematic representation of the intraocular injections of pyrogallol-*O*-sulfate in diabetic and non-diabetic mice, targeting different retinal cells at different stages of DR progression. The expression of proteins having a role in inflammation and neovascularization were assessed by Western blot, two weeks after a single injection, either intravitreous or subretinal.

The animals were randomly assigned for different experimental groups: C57Bl/6 (non-diabetic) sham; C57Bl/6 (non-diabetic) Pyr-s injection; C57BL/-Ins2^Akita^-6J (diabetic) sham; and C57BL/-Ins2^Akita^-6J (diabetic) Pyr-s injection. The experimental protocol for the intraocular injections is schematized below (Figure 12).

Intraocular injections were performed in anesthetized animals with Avertin^®^ ((2,2,2-tribromoethyl alcohol (Sigma-Aldrich, St. Louis, MO, USA) with 2-methyl-2-butanol (Sigma-Aldrich, St. Louis, MO, USA); intraperitoneal injection of 0.5 mL/25 g) and pupils were dilated using 1% tropicamide (Edol, Linda-a-Velha, Portugal). Cornea was superficially anesthetized with Anestocil (Edol, Linda-a-Velha, Portugal) as an eye drop. A 30-gauge needle was used to puncture a hole approximately 1 mm below the limbus in the immobilized animals. The left eye was injected intravitreally or subretinally with 2 µL of Pyr-s at a final concentration of 6.5 µM or saline solution in one eye using a Hamilton syringe 10 µL 701 RN injector at 50 nL/s. The non-injected contralateral eye was used as control. After the injection, gentamicin and dexamethasone were applied in the injected eye to reduce the risk of infection.

No changes in grooming, behavior, food, or water consumption were observed after injection. Additionally, visual inspection of the eyes of animals within the experimental groups revealed no signs of ocular inflammation nor changes in overall health status.

#### 4.7.3. Retina and RPE Protein Extraction

Two weeks after Pyr-s injection, the animals were humanely sacrificed by cervical dislocation and the eyes removed. The neural retina and RPE were isolated, as previously described [51]. Samples were mechanically homogenized in ice-cold RIPA buffer containing a protease inhibitor cocktail and kept on ice for 20 min. The extracts were then centrifuged (20 min, 4 °C, 16.100× *g*). The supernatant was collected, and protein quantified by Braford method, as previously described, with a dilution of 1:10, for Western blot. The samples were stored at −80 °C until use.

### 4.8. Statistical Analysis

Arithmetic means are given with standard error of the mean (SEM) or standard deviation (SD), when more suitable. Statistical analysis was performed using an unpaired *t*-test with or without Welch’s correction, one-way analysis of variance (ANOVA) with Dunnet’s multiple comparison test, or two-way ANOVA followed by the Sidak’s or Bonferroni’s multiple comparison test for multiple comparisons. A value of *p* < 0.05 was considered to be statistically significant, and a trend change in expression was considered when *p*-value was below 0.25, as previously published [12]. 

## 5. Conclusions

In short, our data demonstrate that exposure of RPE cells to the (poly)phenol-derived metabolite pyrogallol-*O*-sulfate effectively decrease the expression of VEGF in cells under high glucose and hypoxia in certain experimental conditions. Additionally, a single intraocular injection of pyrogallol-*O*-sulfate was able to decrease the expression of the microglial marker Iba1 in the diabetic retina earlier in the disease course. Hence, our findings open an opportunity to further explore this metabolite as a therapeutic approach towards inflammation in DR.

## Figures and Tables

**Table 1:** List of primary and secondary antibodies, with the respective technical information, used for detection of proteins of interest by Western blot.

Primary Antibody	Dilution	Reference	Brand
VEGF-A	1:500–1:1000	ab46154	Abcam, Cambridge, UK
PEDF	1:500–1:1000	07-280	Merck Millipore, Burlington, MA, USA
Iba1	1:500	SAB2500041	Sigma-Aldrich, St. Louis, MO, USA
GFAP	1:10,000	ab7260	Abcam, Cambridge, UK
GLUT-1	1:2000	ab32551	Abcam, Cambridge, UK
β-actin	1:5000	A5441	Sigma-Aldrich, St. Louis, MO, USA
Goat anti-mouse igG-HRP	1:5000	sc-516162	Santa Cruz Biotechnology, Dallas, TX, USA
Goat anti-rabbit igG-HRP	1:5000	sc-2004	Santa Cruz Biotechnology, Dallas, TX, USA
Donkey anti-goat igG-HRP	1:5000	sc-2020	Santa Cruz Biotechnology, Dallas, TX, USA

## Data Availability

Not applicable.

## Supplementary Materials

The following are available online at https://www.mdpi.com/article/10.3390/ijms222111402/s1.
