# Supplementary material for: Polyphenol Metabolite Pyrogallol-O-Sulfate Decreases Microglial Activation and VEGF in Retinal Pigment Epithelium Cells and Diabetic Mouse Retina
Source: Int J Mol Sci. 2021 Oct 22;22(21):11402. doi: 10.3390/ijms222111402 (PMC8583739; doi:10.3390/ijms222111402)
Supplement: Supplementary file 1 [file ijms-22-11402-s001.zip › ijms-1404712-supplementary.pdf]

Supplementary Materials

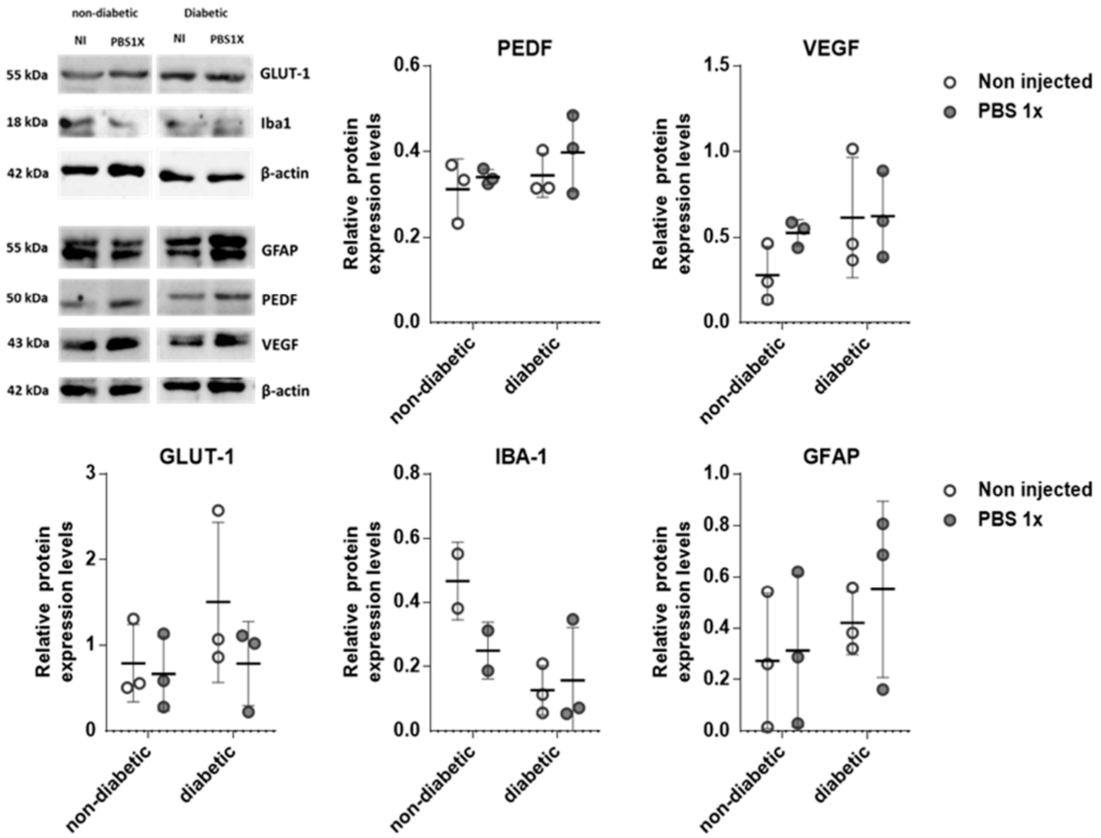

Figure S1 Expression of proteins of interest followed by intraocular injections of vehicle solution (PBS 1x) in diabetic and age-match non-diabetic retinas of animals with 4 month-old. Protein levels were normalized to β-Actin. Data are expressed as mean ± SD (n = 2 to 3 mice/group).

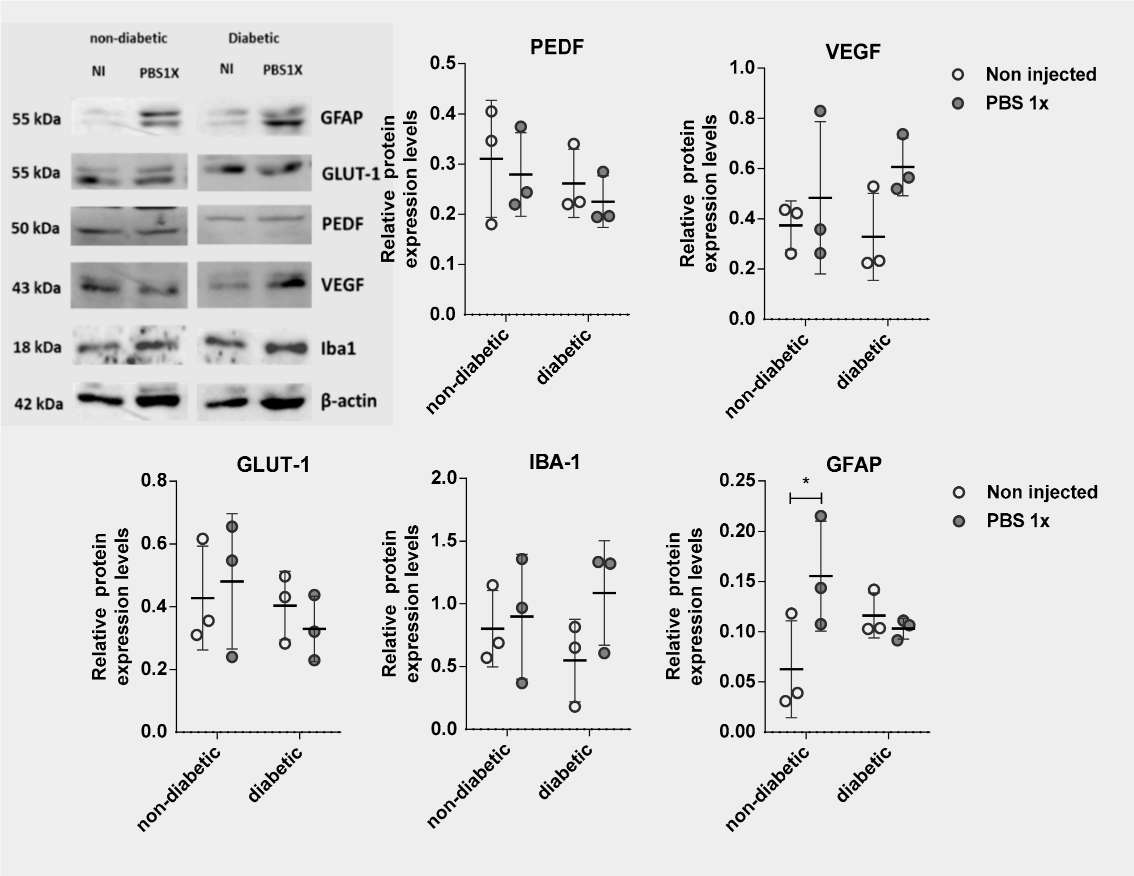

Figure S2 Expression of proteins of interest followed by intraocular injections of vehicle solution (PBS 1x) in diabetic and age-match non-diabetic retinas of animals with 6 month-old. Protein levels were normalized to β-Actin. Data are expressed as mean ± SD (n = 2 to 3 mice/group). * $p < 0.05$ is significantly different compared to contralateral non-injected eye, determined by Two-way ANOVA (age and genotype) with Sidak's multiple comparison test.

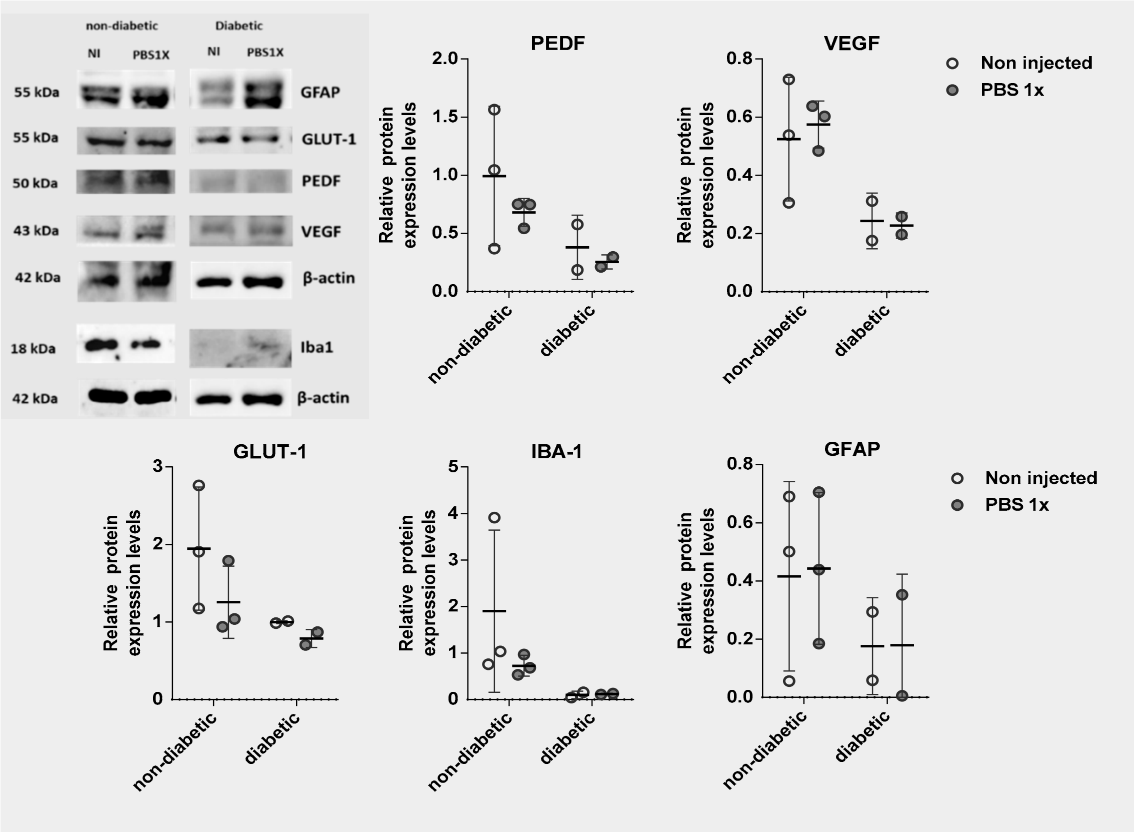

Figure S3. Expression of proteins of interest followed by intraocular injections of vehicle solution (PBS 1x) in diabetic and age-match non-diabetic retinas of animals with 8 month-old. Protein levels were normalized to β-Actin. Data are expressed as mean ± SD (n = 2 to 3 mice/group).

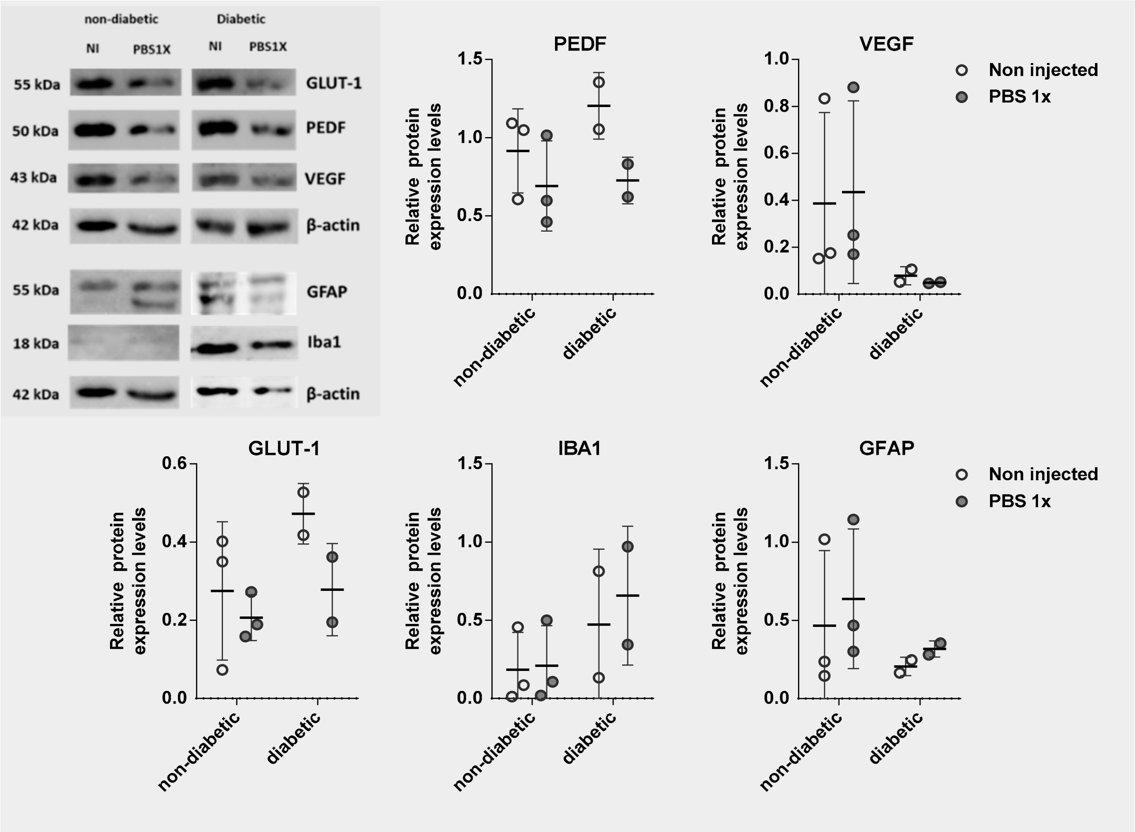

Figure S4. Expression of proteins of interest followed by intraocular injections of vehicle solution (PBS 1x) in diabetic and age-match non-diabetic retinas of animals with 9 month-old. Protein levels were normalized to β-Actin. Data are expressed as mean ± SD (n = 2 to 3 mice/group).

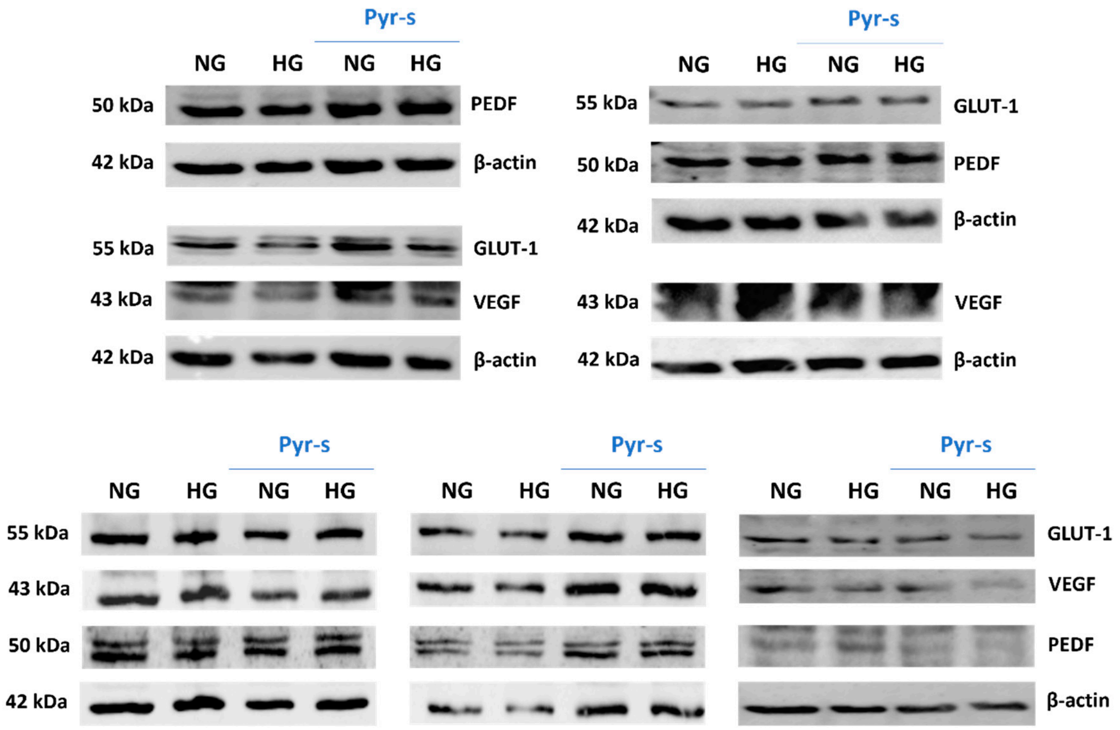

Figure S5. Western blot images showing bands of proteins of interest from RPE cells under normoxia with or without treatment with pyrogallol-*O*-sulfate for 8 hours. These bands were used as controls to normalize bands from cells under hypoxia, exposed to Pyr-s for 8 h upon hypoxic challenge (plotted in Figure 3). $n = 5$.
